# Supplementary material for: Computation is concentrated in rich clubs of local cortical networks
Source: Netw Neurosci. 2019 Feb 1;3(2):384–404. doi: 10.1162/netn_a_00069 (PMC6370472; doi:10.1162/netn_a_00069)
Supplement: Supplementary file 1 [file netn-03-384-s001.pdf]

Faber, S. P., Timme, N. M., Beggs, J. M., & Newman, E. L. (2019). Supporting Information for "Computation is concentrated in rich clubs of local cortical networks." *Network Neuroscience, 3*(2), 384–404. https://doi.org/10.1162/netn_a_00069

SUPPLEMENTAL METHODS

The methods used in this study include many steps. These will be described below in the following order: (1) in vitro data collection; (2) construction of effective connectivity networks; (3) quantification of neural computation; (4) rich club detection in networks of effective connectivity; (5) quantification of the relationship between synergy and rich clubs; (6) control analyses of the synergy-rich club relationship; (7) alternative approaches for quantifying neural computation; and (8) consideration of neuron auto-prediction.

In vitro **data**

To study the relationship between neural computation and topological measures of networks of spiking neurons, we analyzed data collected *in vitro*. Data were spontaneously spiking organotypic cultures of mouse somatosensory cortex obtained from postnatal Day 6 to 7 Black 6 mouse pups (RRID:Charles_River:24101632, Harlan) according to Tang et al., 2008 (Ito et al., 2014). All animal tissue samples were prepared according to guidelines from the National Institutes of Health and all animal use procedures were approved by the Indiana University Animal Care and Use Committee as well as the Animal Care and Use Committee at the University of California, Santa Cruz. Spontaneous (as opposed to stimulus-driven) spiking activity in the cultures was recorded at a high temporal resolution of 50 μs, between 2 and 4 weeks after culture preparation, using a 512-microelectrode array (Litke et al., 2004). Array electrodes were flat, 5 μm in diameter and arranged in a triangular lattice with an interelectrode distance of 60 μm. This spacing means that the spiking of most cells is picked up by multiple sites and there are few gaps where cells are too far from electrodes to be recorded. The full array allowed for a total recording area of approximately 0.9 mm by 1.9 mm. This preparation and recording method enabled the isolation of large numbers of neurons (an average of 309 cells per recording in 25 hour-long recordings) at high temporal resolution, beyond what can currently be done in any *in vivo* setup. Crucially, the temporal resolution of this method was small enough to resolve synaptic delays of 1-20 ms typically found in cortex (Mason et al., 1991; Swadlow, 1994).

Once the data were collected, spikes were sorted using a PCA approach based on waveforms detected at seven adjacent electrodes (Ito et al., 2014; Litke et al., 2004; Timme et al., 2014). This process yielded a single set of spike times for each isolated neuron. Neurons that spiked fewer than 100 spikes during the hour long recording were removed from the analysis. Spike trains were then used to build networks.

Effective connectivity network construction

Because neural computation is fundamentally a dynamic process, we focused on examining networks of effective connectivity. In these networks, connections represent a predictive relationship between the firing of two different neurons. Note, effective connectivity differs from structural connectivity (synapses or gap junctions between neurons) and functional connectivity (e.g., cross-correlations between neuronal time series). Here, effective connections represent directed information transfer between neurons.

Networks of effective connectivity, representing global activity in recordings, were constructed according to Timme et al (2014, 2016) using a measure from information theory known as transfer entropy (TE; Schreiber, 2000). TE was selected for its ability to detect nonlinear interactions and deal with discrete data, such as spike trains. To capture neuron interactions at timescales relevant to synaptic transmission (14 ms; Mason et al., 1991; Swadlow, 1994), multiple windows are used to improve the sensitivity to functional interactions across these delays. spiking data was binned at three logarithmically-spaced bin sizes (1, 1.6 and 3.5 ms) and TE was computed at delays (0-3 bins, for bins of size 1 and 1-4 bins for bins of size 1.6 and 3.5 ms) corresponding to synaptic delays, as in Timme et al. (2014, 2016). Thus, we computed TE at three timescales, 0.05–3 ms, 1.6–6.4 ms and 3.5–14 ms. Timescales were purposefully designed to be overlapping so that no interactions were neglected. See Figure 1 for an overview of the binning structure used in TE calculations.

TE quantifies an effective connection from neuron J to neuron I by measuring how much information the past state of the neuron J time series (J_{t-1}) produces regarding the current state of the neuron I time series (I_t), beyond what is provided by the past state of the neuron I time series (I_{t-1}). Here, time series are binary spike trains for neurons I and J, containing 0 for time bins in which the neuron did not spike and 1 for time bins in which it did spike. Generally, the TE from neuron J to neuron I is computed as:

$$TE_{J \to I} = \sum_{i_t, i_{t-1}, j_{t-1}} p(i_t, i_{t-1}, j_{t-1}) \log\left(\frac{p(i_t|\, i_{t-1}, j_{t-1})}{p(i_t|i_{t-1})}\right) \tag{1}$$

The probabilities in Eqn. 1 are computed by counting the number of occurrences of all possible combinations of spiking and not spiking in the i_t, i_{t-1} and j_{t-1} time bins (of the I_t, I_{t-1} and J_{t-1} time series) for all bins making up the hour-long recording.

Because we wanted to consider interactions at three timescales associated with synaptic transmission, we included a delay between the past and future states of the neurons so that i_{t-1} became i_{t-d} and j_{t-1} became j_{t-d}. Additionally, in order to ensure overlapping timescales, we combined the i_{t-d} and j_{t-d} bins with their previous time bins, such that a spike in either or both time bins corresponded to a state of 1 while no spikes in either time bin corresponded to a state of 0 (See Figure 1 for binning structure). Denoting these new bins as i'_{t-d} and j'_{t-d} gives a slightly different form for TE:

$$TE(d)_{J \to I} = \sum_{i_t, i'_{t-d}, j'_{t-d}} p(i_t,\, i'_{t-d},\, j'_{t-d}) \log\left(\frac{p(i_t|\, i'_{t-d},\, j'_{t-d})}{p(i_t|\, i'_{t-d})}\right) \tag{2}$$

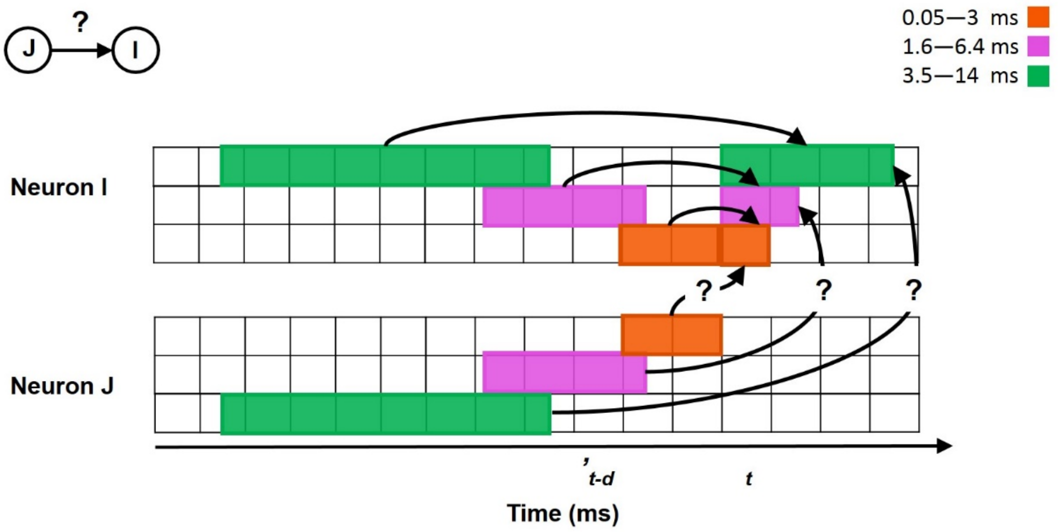

Figure 1. *Overview of time series binning structure used in transfer entropy calculations.* Transfer entropy was used to quantify a directed, functional connection from neuron J to neuron I which represents how well the current state (t) of neuron I can be predicted by the past state ('t-d) of neuron J, beyond what is known from the past state of neuron I itself. Three timescales were considered, each with corresponding delays (d). Timescales considered transfer entropy from 0.05—3 ms, 1.6—6.4 ms, and 3.5—14 ms.

To cast TE in terms of the percentage of the receiver neuron's capacity that can be accounted for by the transmitting neuron, rather than it representing the amount of information being transmitted from transmitter to receiver, we normalized TE by the entropy of the receiver neuron via:

$$TE_{Norm}(d)_{J \to I} = \frac{TE(d)_{J \to I}}{-\sum_{i_t} p(i_t) \log\left(p(i_t)\right)} \tag{3}$$

Computing (normalized) TE in this way between all pairs of binned neuronal time series results in a time-scale dependent, weighted, directed network. Networks are weighted because some pairs of neurons fire more frequently and reliably at certain delays than others, and they are directed because a predictive, statistical relationship that exists from neuron J to neuron I, may not exist from neuron I to neuron J. Each element a_{ij} in the TE matrix is the TE value from the ith to the jth neuron. TE values of zero denote the absence of an effective connection between the two neurons, while TE values greater than zero represent the weighted strength of the effective connection between the two neurons.

To determine the significance of network connections (TE values), TE values were computed for 5000 pairs of jittered spike trains. TE values which were larger than 99.9% of jittered values were considered significant, corresponding to a p-value of less than 0.001. Computing significant, normalized TE values for 25 recordings at three timescales, resulted in 75 full networks.

To ensure that the detection of rich clubs was not biased by the spatial sampling of the recording apparatus, we compared the distances between rich club neurons (defined as the top 30% of neurons in a network) to the distances between all neurons in the network. We found that there were no significant differences between the two distributions of distances (KS tests revealed that 75 out of 75 networks had distributions that were not significantly different at the $\alpha = 0.01$ level; Figure 2).

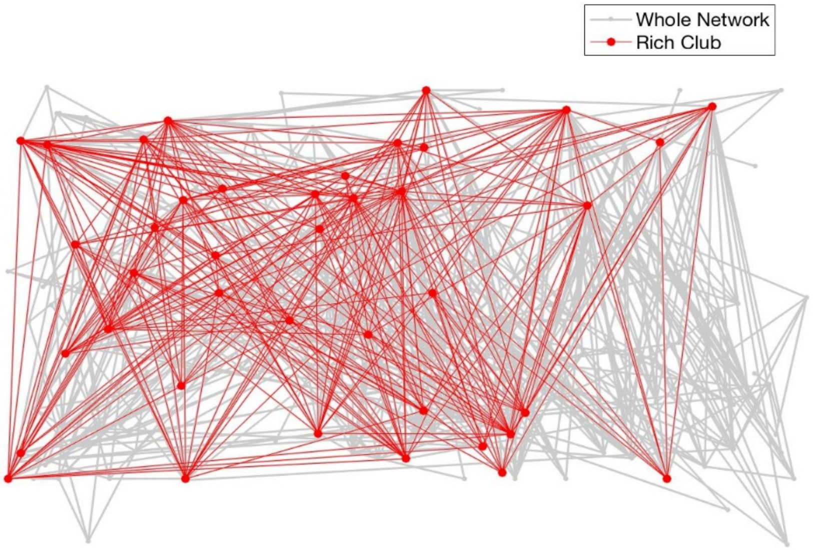

Figure 2. ***Sp****atial distribution of rich club neurons is not significantly different from the spatial distribution of the full network.* Spatial distribution of rich club neurons relative to all neurons in a representative network.

To be confident that our timescales captured the peak of information processing in our networks, we calculated TE at delays other those analyzed here for two representative networks. First, spike trains were binned at 1 ms. Then TE was calculated at multiple delays ranging from 0 to 501 ms, in steps of 3 ms, for all existing pairs of significant connections in the network. We found that TE tended to peak in the 1-14 ms delay range for most connections (Figure 3). In fact, for 87.3% of pairs on average, the peak of TE occurred at between 1 and 14 ms.

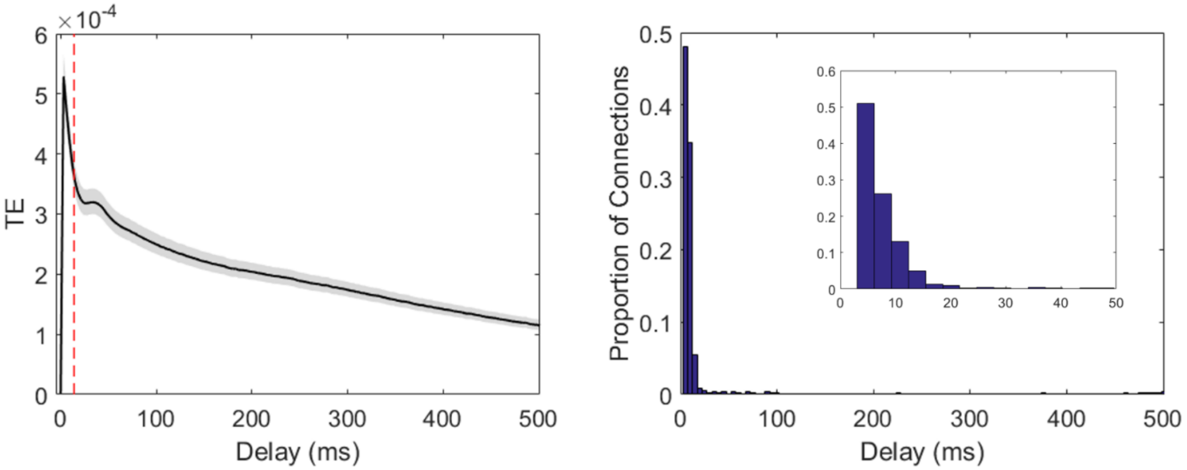

Figure 3. *TE peaks between 1-14 ms.* Mean distribution of TE over time for all connections from two representative networks. Left: The black line shows the mean TE over all connections from two representative networks. The shaded region shows the 95% confidence interval. The vertical dashed red line indicates the upper bound of the timescales analyzed in the manuscript. Across connections, the peak TE occurs below this bound at short latencies. Right: Histogram of the delay to the maximum TE over connections. The height of each bar shows the proportion of connections for which the peak TE was found to occur at the delay indicated along the x-axis. Most connections had max TE at short delays as shown in the inset panel which zooms in to the first 50 ms of the x-axis. These plots show that most connections had a peak TE at less than 14 ms.

Quantification of neural computation

Computation by neurons receiving inputs from two other neurons in these networks was quantified following the partial information decomposition (PID) from Williams and Beer (2010). This method was used as it is currently the only method capable of quantifying how much computation occurs in an interaction in which three variables predict a fourth as done here (the future state of the receiver is predicted from the past state of the receiver and two other transmitters). The PID allows multivariate TE to be separated into distinct information components, one of which is a measure of neural computation termed synergy. The general form of the decomposition of multivariate TE between three neuronal time series, with two transmitter neurons, J and K, each sending a single input to one receiver neuron, I, can be expressed as (Figure 4):

$$TE(\{J,K\} \rightarrow I) = \text{Synergy}(\{J,K\} \rightarrow I) + \text{Unique}(K; J \rightarrow I) + \text{Unique}(J; K \rightarrow I) + \text{Redundancy}(\{J,K\} \rightarrow I) \quad (4)$$

where $\{J,K\}$ is a vector of the combined J and K time series. Similarly, we can express the decomposition of bivariate TE from neuron J to I and neuron K to I as (Figure 4):

$$TE(J \rightarrow I) = \text{Unique}(K; J \rightarrow I) + \text{Redundancy}(\{J,K\} \rightarrow I) \quad (5)$$

and

$$TE(K \rightarrow I) = \text{Unique}(J; K \rightarrow I) + \text{Redundancy}(\{J,K\} \rightarrow I) \quad (6)$$

In Equations 4-6, all terms are quantified in units of bits (see Williams and Beer 2010, 2011 for a full description of these terms). The unique terms correspond to the information provided by that time series alone (either the J or the K time series) about the current state of I. The redundant term represents the overlapping information provided by time series J and K about the current state of I. Notice, in Equations 5 and 6, that although TE is only dependent on the two time series that are directly interacting (either J and I, or K and I), because J and K are both interacting with the same time series, their unique interactions are influenced by each other. Thus, the unique information provided by one of these time series is dependent on the other. In other words, because J and K provide some redundant (overlapping) information about I, J influences how much information K provides uniquely versus redundantly about I. Likewise, K influences how much information J provides uniquely versus redundantly about I.

The synergistic term in Equation 4 is the additional information (beyond the unique and redundant information) that is processed by the receiver (I) based on the non-overlapping information from both inputs (J and K) occurring simultaneously. Thus, synergy is a proxy for the non-linear computation which takes information from two sources and combines them in some way to generate a unique output.

To calculate synergy, note that Equation 4 can be rewritten as:

$$\text{Synergy}(\{J,K\} \to I) = TE(\{J,K\} \to I) - TE(J \to I) - TE(K \to I) + \text{Redundancy}(\{J,K\} \to I) \tag{7}$$

by substituting Equations 5 and 6 and solving for synergy. Notice that we can compute all TE terms in Equation 7 via Equation 1. This leaves only the Redundancy term to be computed. Fortunately, a method for measuring this term has been provided by Williams and Beer (2010, 2011), who define redundancy in terms of a quantity titled the minimum information $I_{\min}$:

$$\text{Redundancy}(\{J,K\} \to I) \stackrel{\text{def}}{=} I_{\min}(I_t; J_{t-1}K_{t-1} | I_{t-1}) = \sum_{i_t} p(i_t) \min_{R \in \{J_{t-1}, K_{t-1}\}} I_{spec}(I_t = i_t; R | I_{t-1}) = \sum_{i_t} p(i_t) \min_{R \in \{J_{t-1}, K_{t-1}\}} [I_{spec}(I_t = i_t; R, I_{t-1}) - I_{spec}(I_t = i_t; I_{t-1})] \tag{8}$$

where the specific information I_{spec} is defined as:

$$I_{spec}(I_t = i_t; R, I_{t-1}) = \sum_{r,\, i_{t-1}} p(r, i_{t-1} | i_t) \log\left(\frac{p(r, i_{t-1}, i_t)}{p(r, i_{t-1}) p(i_t)}\right) \tag{9}$$

and

$$I_{spec}(I_t = i_t; I_{t-1}) = \sum_{i_{t-1}} p(i_{t-1} | i_t) \log\left(\frac{p(i_{t-1}, i_t)}{p(i_{t-1}) p(i_t)}\right) \tag{10}$$

Thus, redundancy is the minimum information provided by J or K about each state of I, averaged over all possible states. In other words, redundancy is the minimum overlapping information (the shared information) that the past states of J and K provide about the current states of I. Redundancy was calculated via Equations 9 and 10. Finally, synergy was calculated via Equation 7. Computing synergy for all possible triads (for each neuron that received at least two inputs, all possible groupings of two input neurons and the receiver were considered) in all networks yields a single synergy value per triad. We then normalized synergy values by dividing by the entropy of the future state of I, as done in Equation 3.

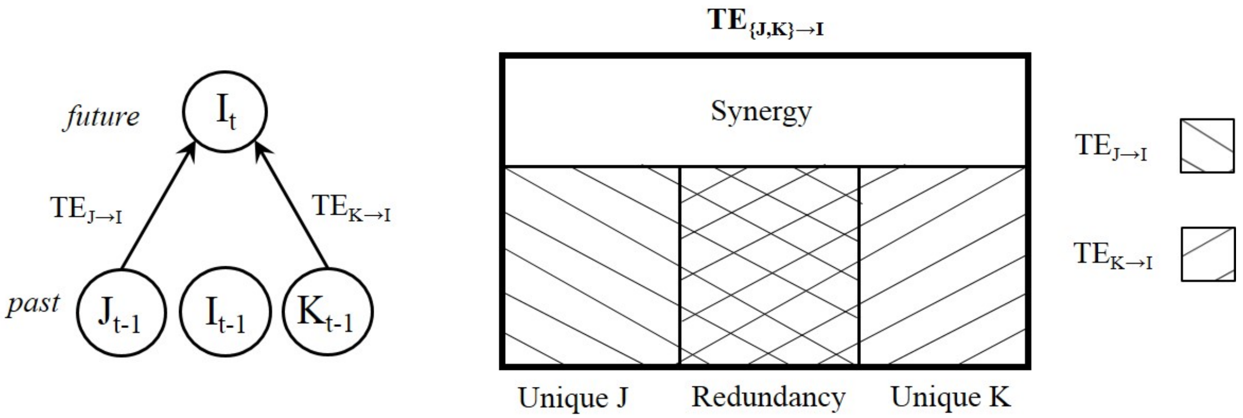

Figure 4. *The Partial Information Decomposition.* In this study, we analyzed two-input computations which were determined using the Partial Information Decomposition to dissect multivariate transfer entropy (occurring among three neurons, with two transmitter neurons each sending significant information to a receiver neuron) into synergistic, redundant, and unique information terms. The synergistic information component was used to represent the amount of computation carried out by the receiver.

Although there are other methods for calculating synergy (Bertschinger et al., 2014; Pica et al., 2017), we chose this measure because it is capable of detecting linear and nonlinear interactions and it is currently the only measure which can quantify how much synergy occurs in an interaction in which three variables (here, receiver past and pasts of the two transmitters) predict a fourth. Note, we chose not to consider higher order synergy terms, for systems with more than two transmitting neurons, due to the increased computational burden it presented (the number of PID terms increases rapidly as the number of variables increases). However, based on bounds calculated for the highest order synergy term by Timme et al. (2016), it was determined that the information gained by including an additional input beyond two either remained constant or decreased. Thus, it was inferred that lower order (two-input) computations dominated.

It is important to re-emphasize two things here. First, transmitter neurons and receiver neurons differ in terms of how they are defined. Transmitter neurons are required to have at least one outgoing connection (but not necessarily any incoming connections) and receiver neurons are required to have at least two incoming connections (but not necessarily any outgoing connections). Second, synergy occurs at the receiver neuron (where the two input signals are integrated). Thus, transmitters can be thought of as contributing to computation which occurs at the receiver neuron.

Rich club detection

To examine the relationship between rich clubs and computation in our networks, we identified the weighted rich clubs in each recording. Weighted rich clubs were identified using a modified version of the rich_club_wd.m function from the Matlab Brain Connectivity toolbox (Rubinov and Sporns, 2010; van den Heuvel and Sporns, 2011), adapted according to Opsahl et al. (2008) to compute weighted rich clubs. Briefly, this algorithm computes weighted rich-club coefficients as follows. For a given recording, a richness parameter (r), defined as the sum of the weights (incoming and outgoing), was computed for all neurons. Then, for every value of r observed in a recording, a weighted rich club coefficient is computed. A rich club coefficient is computed for the kth value of r as follows:

$$\Phi^w(r_k) = \frac{\sum_{i_{r \geq r_k}} \sum_{j_{r \geq r_k}} TE_{\{i,j\}}}{\sum^n TE^{rank}} \tag{6}$$

Here, the numerator is the amount of information transfer between neurons with r greater than or equal to r_k, computed as the sum of the TE value between these neurons. The denominator is the maximum amount of information transfer that could have been observed among the neurons with r greater than or equal to r_k. This is computed as the sum of the largest n weights in the network where n is the number of edges found between neurons with r greater than or equal to r_k. The resulting ratio approaches one when the strongest connections connect the neurons that transfer the most information (i.e., richest neurons).

To establish the existence of a significant rich club at a given threshold r_k, we computed the ratio between the observed $\Phi^w(r_k)$ and the distribution of those observed when the edges of the network were shuffled. Shuffles were performed according to the methods of Maslov and Sneppen (2002). Briefly, this method randomly selects two edges (e.g., A→B and C→D) and randomly swaps either the sender or the receivers of the edges (A→D and C→B). Such rewiring only takes place if the newly created edges did not already exist in the network. To shuffle a network, the swapping process is repeated four times the number of edges in the network.

For each network, the rich club coefficients from shuffled versions of the network ($\Phi^w_{shuffled}$) were computed from 500 shuffled variants of the network. The mean coefficient for each threshold r_k across shuffles was then used to normalize the observed coefficients, as follows:

$$\Phi^w_n(r_k) = \frac{\Phi^w_{observed}(r_k)}{|\Phi^w_{shuffled}(r_k)|} \tag{7}$$

The resulting normalized coefficient, Φ^w_n, reflects how many times greater the observed coefficients are than the expected values given the distribution of edge weights observed in a given network. The significance of a given normalized coefficient was established by computing the associated p-value as the number of coefficients from shuffled networks that exceeded the observed coefficient and dividing by the number of shuffles (500). We defined p-value ≤ 0.01 as significant.

Quantification of the synergy-rich club relationship

The relationship between synergy and rich clubs was performed using two approaches. In the first approach, we compared the amount of computation inside versus outside of the rich club by randomly selecting a single, significant representative rich club for each network and asking what the expected synergy-per-triad was for triads with receivers inside versus outside of the rich club. Given the risk for sampling bias introduced by the selection of representative rich clubs in the first approach, we also pursued a second approach that quantified the relationship between synergy and the rich club, at all possible thresholds. That is, for each network, we sorted neurons from strongest to weakest, and then cumulatively recruited neurons into the "rich club" one at a time. For this second approach, we computed the amount of computation inside and outside of the rich clubs

defined at all possible thresholds for each network. The results were then aggregated across networks by aligning coefficients based on the percentage of the network included in the rich club. The above approaches were also used to compare the computation ratio, or the ratio of computation to propagation (summed triad TE), inside versus outside the rich club.

In addition to the above analyses which considered the position of the receiver with respect to the rich club, we also calculated the expected synergy found in all possible interactions of triads with respect to the rich club for all networks (see Figure 5). This was done to achieve a more detailed understanding of the relationship between triads, synergy, and rich clubs.

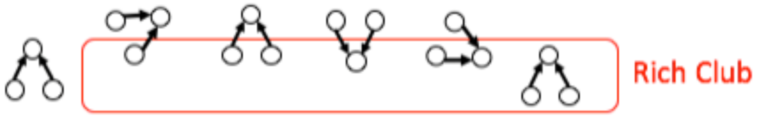

Figure 5. Schematic of possible configurations of synergistic triads interacting with the rich club. In order to quantify the amount of computation that takes place within the rich club and to determine how the amount of computation depends on the interaction between synergistic triads and the rich club, we considered all ways in which synergistic triads could interact with the rich club. From left to right these include: no triad nodes or edges participate in the rich club, a single transmitter (arrow pointing away) is in the rich club, both transmitters are in the rich club, only the receiver (both arrows pointing toward) is in the rich club, a transmitter-edge-receiver combination is in the rich club, and finally, the entire connected triad is in the rich club.

Control analyses of the synergy-rich club relationship

<u>Control analysis 1:</u> In order to account for the fact that networks and rich clubs were defined by TE, a fact which may trivialize the result of greater synergy in rich clubs, we tested the relationship between synergy and the rich club after permuting the spike times of one transmitter in a triad with "bonafide" synergy. Here, a triad has bonafide synergy if the total information gained about the future state of neuron I is greater than the sum of the information provided by neuron J and K in total (assuming zero redundancy, thus making this maximally conservative) which is expressed as $TE(\{J,K\} \rightarrow I) > TE(J \rightarrow I) + TE(K \rightarrow I)$. Spike times were permuted by first identifying all time bins for which R_p and R_f (R_p = receiver state in the past; R_f = receiver state in the future) are in one of the four basic configurations { [0,0], [0,1], [1,0], [1,1] }. For each configuration, we shuffled the spike times of the transmitter. By shuffling within configurations of the receiver state we preserve the transfer entropy from the transmitter to the receiver, but destroy the relationship between transmitters and therefore leading to a different value of synergy. We repeated this shuffling procedure 200 times per triad, generating a null distribution of synergy, for all triads with bonafide synergy in all 75 networks. We then asked whether the null synergy was greater in the rich club compared to outside. We determined that there is significantly greater median null synergy in the rich club for the 1.6–6.4 ms timescale 0.0016 (Zs.r.= 4.34, n = 25 networks, p = 1.39x10-5) and the 3.5–14 ms timescale 0.002 (Zs.r.= 4.2, n = 25 networks, p = 2.54x10-5), but not for the 0.05–3 ms timescale 2.5x10-5 (Zs.r. = 0.46, n = 25 networks, p = 0.65). We then compared these differences to those obtained from the observed synergy and found that there is significantly greater synergy in rich clubs than what would be expected by chance (Zs.r. = 5.76, n = 75 networks, p = 8.6x10-9). The empirically observed synergy values were significantly greater in the rich club in 88% (66 out of 75) of the networks. Converting the observed synergy into a z-score based on the distribution of null synergy values results in a median z-score of 13.31 over all networks (25.8, 12.03, and 13.5 for timescales 0.05–3 ms, 1.6–6.4 ms and 3.5–14 ms,

respectively). The results of this analysis demonstrate that the computation observed in the rich club is not a simple consequence of the magnitude of the TE values that comprise the rich clubs in these networks.

Control analysis 2: To demonstrate that the result of synergy in the rich club could not have been explained by simple correlations between incoming weight of the receiver and synergy, we asked if there is greater synergy in the rich clubs after the correlation between connection strength and synergy has been regressed out. To do this, we performed a regression between summed incoming connection strength and synergy across triad receivers for a given network and then collected the residual synergy for each triad after accounting for the summed incoming connections. We then asked if the residual synergy values were still significantly greater in the rich club than outside and found that they were ($Z_{s.r.}$ = 6.29, n = 75 networks, p = 3.24x10^{-10}).

Control analysis 3: To illustrate that outgoing connections are not synonymous with synergy, and to be sure that potential correlations between the strength of outgoing connection and synergy did not bias the results reported here, we calculated correlations between summed outgoing connection strength of the receiver and synergy for all triads in all networks. We found that synergy and outgoing connection strength are not strongly related. Synergy was slightly negatively correlated with outgoing connection strength at the longest (3.5–14 ms) timescale (ρ = -0.07, $Z_{s.r.}$ = -3.54, n = 25 networks, p = 0.0004). The sign of the correlation between outgoing weight and synergy was slightly positive at the shorter timescales (ρ = 0.06, $Z_{s.r.}$ = 1.98, n = 25 networks, p = 0.048 for the 0.05–3 ms timescale; and, ρ = 0.01, $Z_{s.r.}$ = 0.2, n = 25 networks, p = 0.84 for the 1.6–6.4 ms timescale). However, when all networks were considered together, the distribution of correlation values was centered on zero ($Z_{s.r.}$ = -0.8, n = 75 networks, p = 0.42). The only of these timescales which had a strong relationship between weight and synergy was the 3.5–14 ms timescale, but the relationship was negative, which would work against the pattern of results reported here that synergy is greatest in rich clubs. In addition to this, we calculated correlations between summed incoming and outgoing connection strengths of the receivers. Similarly, we did not find evidence of a clear relationship between these correlations and the result of synergy in the rich. The median correlation values for timescales 0.05–3 ms, 1.6–6.4 ms and 3.5–14 ms were ρ = 0.33 ($Z_{s.r.}$ = 3.7, n = 25 networks, p = 0.0002), ρ = 0.07 ($Z_{s.r.}$ = 2.25, n = 25 networks, p = 0.02), and ρ = -0.13 ($Z_{s.r.}$ = -3.13, n= 25 networks, p = 0.002), respectively. The fact that the sign and strength of correlations between summed incoming and outgoing connections strengths across triads did not closely track difference in synergy density inside versus outside the rich club suggests this was not a confounding variable.

Alternative approaches to standard PID

To ensure that our findings were not method-dependent, we performed additional analyses which implemented alternative methods for calculating synergy. First, we considered the effect of calculating the lower bound on synergy, which we refer to as "bonafide" synergy. Second, we considered an approach which, instead of PID, calculates neuron transfer functions according to the method proposed by Chichilnisky (2001). This method begins with the calculation of each neuron's spike-triggered average (STA), which is the average pattern of input spikes preceding a spike in the cell (i.e. the sum of the inputs i preceding each spike, divided by the total number of spikes f) for a specific delay d:

$$\text{STA} = \frac{\sum_{t=1}^{d} i_t f_t}{\sum_{t=1}^{d} f_t} \quad (8)$$

Here, we considered a delay of 1-14 ms. In other words, we looked at input patterns occurring anywhere from 14 to 1 ms before the spike of each neuron. To determine each neuron's response based on its STA, we next calculated a *generator signal*: $g_t = a \cdot s_t$, which is a linear combination of the input spikes at a particular time, and then examined the average spike count in time bins with approximately equivalent generator signals (Chichilnisky, 2001). This gave us a neuron transfer function in the form of probability of spiking vs. generator signal (number of inputs). Because this relationship can be linear or nonlinear, we then fit each neuron's transfer function with both a linear and nonlinear (sigmoidal) fit, and calculated the sum of squared errors for each fit. We then computed the ratio of the sum of squared errors for the sigmoidal fit to sum of squared error for the linear fit and used a median split to classify neurons as having either linear or nonlinear transfer functions. Neurons whose sum of squared errors ratio was greater than the median were classified as linear, whereas those whose sum of squared errors ratio was less than the median were classified as nonlinear.

To examine how this approach relates to PID, we compared the expected synergy for neurons with nonlinear versus linear transfer functions across all networks. To parallel our synergy-rich club analysis, we also compared the concentration of neurons with nonlinear transfer functions inside and outside the rich club. That is, we computed the percentage of rich club and non-rich club neurons that had nonlinear transfer functions.

Consideration of neuron auto-prediction

A further analysis was done to assess the risk posed by the possibility that our use of small windows when defining the past state of a receiver may underestimate neuron auto-prediction. Such underestimation could result in inflated TE and synergy values. To address this, we performed an analysis, similar to Nigam et al., 2016, in which we jittered transmitter neuron spike trains with respect to receiver neuron spike trains at short timescales, thereby disrupting short timescale relationships between the neurons but leaving longer timescale statistics intact, and retaining the auto-prediction of the neuron. We calculated new transfer entropy values as $TE_{new} = TE_{observed} - TE_{jittered}$, where $TE_{jittered}$ was the mean of TE values obtained from 100 different jitters. When jittering, spike times were shifted randomly within a 3 bin window. Synergy values were calculated in a corresponding fashion, by jittering both transmitters with respect to the receiver, and subtracting off the mean of the jittered synergy values. We then repeated the core analyses of synergy in the rich club with these updated values (results shown in Supplemental Figure 11).

SUPPLEMENTAL RESULTS

The current study investigated the relationship between synergy and rich clubs at multiple timescales relevant to synaptic connectivity. Although results have been pooled in the manuscript, here we show them for each timescale separately. First, we show the existence of rich clubs across

networks at each timescale (Figure 6). Second, we show that there is greater synergy in rich clubs across networks at each timescale (Figure 7) and that mean network synergy correlates with normalized rich club coefficient across networks at each timescale (Figure 8). Third, we show that alternative methods to the original PID produce qualitatively similar results of synergy in the rich club. Specifically, we use an alternative implementation of PID (Figure 9), as well as a non-information theoretic approach to measuring neural computation (Figure 10). Finally, we show the results of the bootstrap method which updates TE and synergy values to reflect variance accounted for by auto-prediction (Figure 11). The findings of these analyses agree with previous results that synergy is elevated in the rich club.

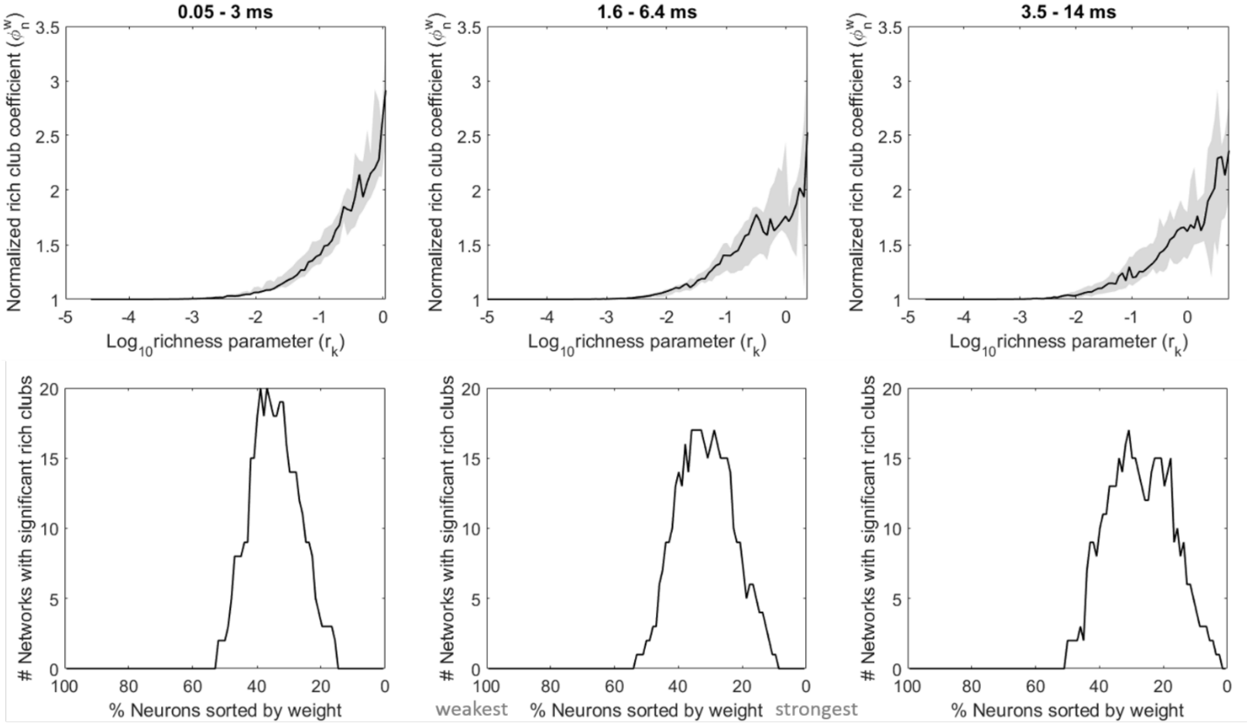

Figure 6. *Network rich clubs.* Aggregate rich club curves for all 25 networks at each timescale. The number of significant rich clubs that were found at each of the possible subnetworks (% neurons sorted by weight) across all networks is also shown.

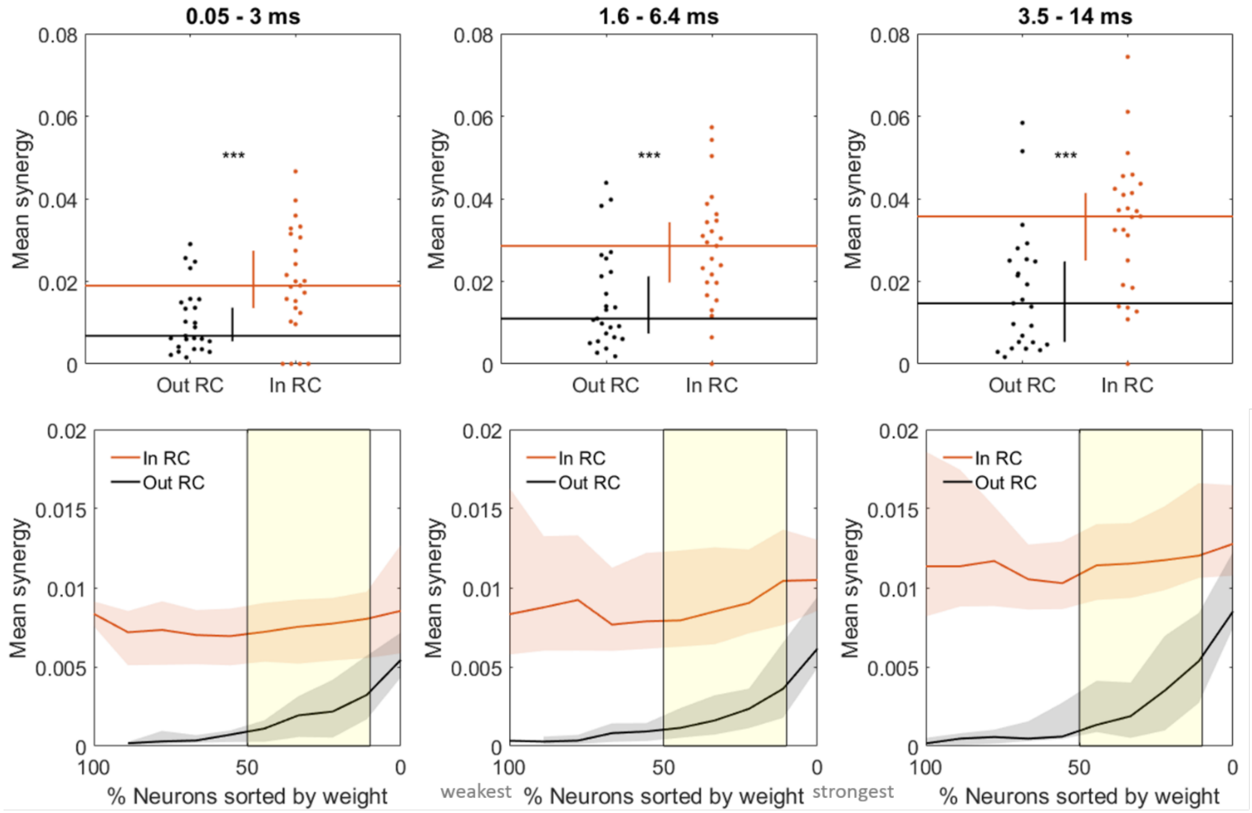

Figure 7. *Synergy in rich clubs.* Synergy is greater in rich clubs at all timescales. This is shown for a significant, representative rich club (top row), as well as at all significant rich club levels (yellow shaded region, bottom row). *$***P < 1 \times 10^{-6}$*

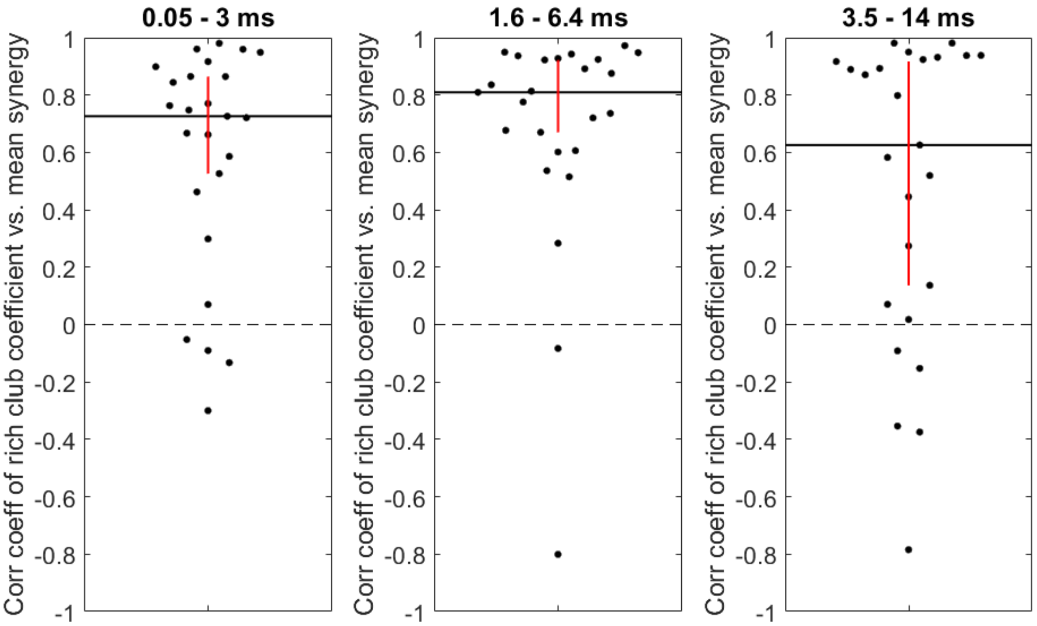

Figure 8. *Mean synergy correlates with normalized rich club coefficient.* Correlation values shown for each timescale separately.

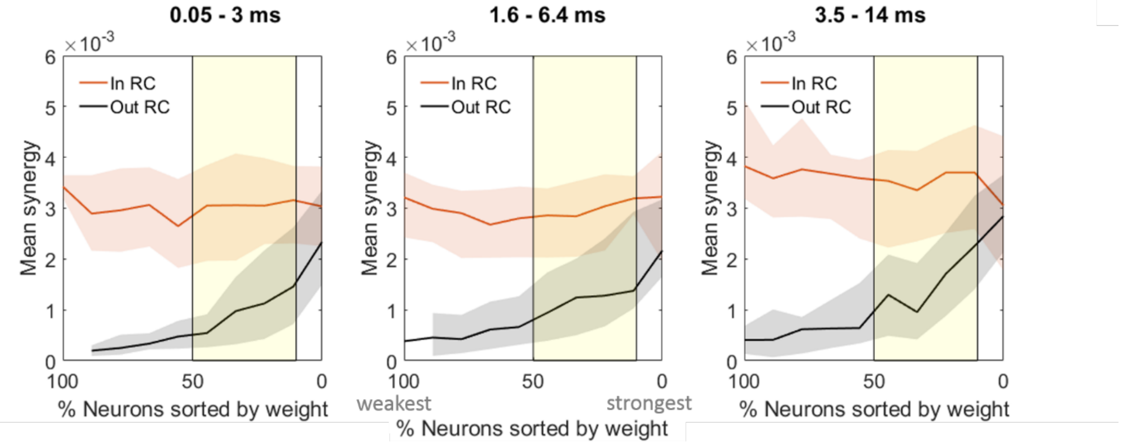

Figure 9. *Results of "bonafide" synergy analyses.* Mean "bonafide" synergy is significantly greater in the rich club at all significant rich club levels (yellow shaded region), at all timescales.

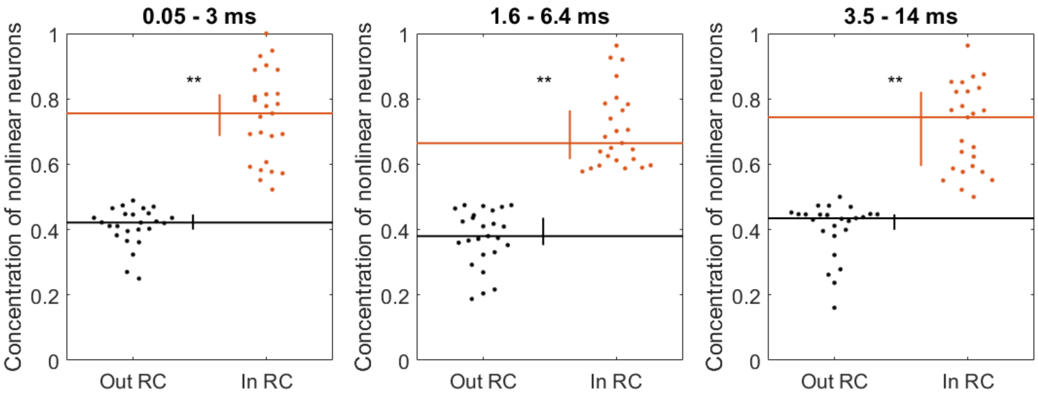

Figure 10. *Concentration of nonlinear neurons is highest in the rich club.* The concentration of nonlinear neurons, according to the classification from the Chichilnisky analysis, is significantly larger in the rich club, for all timescales. $**P < 1 \times 10^{-4}$

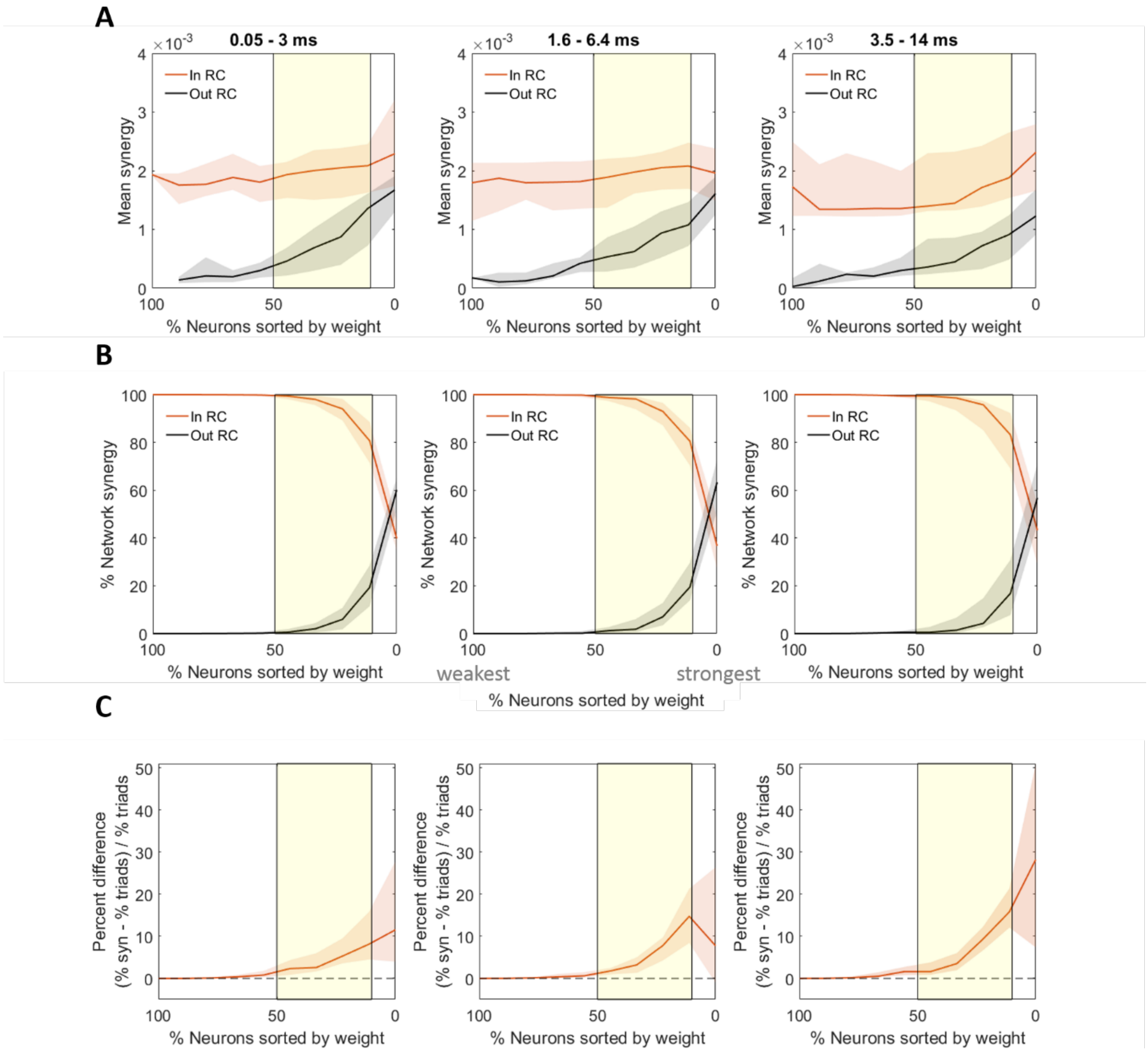

Figure 11. *Synergy is highest in the rich club after subtracting TE and synergy values that result from jittered spike trains..* (A) Rich club neurons have greater synergy at all significant rich club levels (signrank: $p = 0.002$, $z = 2.88$). (B) The percentage of total network synergy is higher for neurons in the rich club, at significant rich club levels (signrank: $p = 0.004$, $z = 2.66$). (C) The percentage of total network synergy accounted for by rich club neurons is significantly greater than expected given the percentage of triads in the rich club, at all significant rich club levels (signrank: $p = 0.004$, $z = 2.66$). Significant rich club levels indicated by yellow shaded region.
